# It’s Not Easy Being Green: A Narrative Review on the Microbiology, Virulence and Therapeutic Prospects of Multidrug-Resistant *Pseudomonas aeruginosa*

**DOI:** 10.3390/antibiotics10010042

**Published:** 2021-01-04

**Authors:** Payam Behzadi, Zoltán Baráth, Márió Gajdács

**Affiliations:** 1 Department of Microbiology, College of Basic Sciences, Shahr-e-Qods Branch, Islamic Azad University, Tehran 37541-374, Iran; p.behzadi@qodsiau.ac.ir; 2 Department of Prosthodontics, Faculty of Dentistry, University of Szeged, Tisza Lajos körút 62-64, 6720 Szeged, Hungary; barzol34@gmail.com; 3 Institute of Medical Microbiology, Faculty of Medicine, Semmelweis University, 1089 Budapest, Hungary; 4 Department of Pharmacodynamics and Biopharmacy, Faculty of Pharmacy, University of Szeged, 6720 Szeged, Hungary

**Keywords:** *Pseudomonas aeruginosa*, MDR, colistin, cephalosporin, carbapenem, virulence, biofilm, therapy

## Abstract

*Pseudomonas aeruginosa* is the most frequent cause of infection among non-fermenting Gram-negative bacteria, predominantly affecting immunocompromised patients, but its pathogenic role should not be disregarded in immunocompetent patients. These pathogens present a concerning therapeutic challenge to clinicians, both in community and in hospital settings, due to their increasing prevalence of resistance, and this may lead to prolonged therapy, sequelae, and excess mortality in the affected patient population. The resistance mechanisms of *P. aeruginosa* may be classified into intrinsic and acquired resistance mechanisms. These mechanisms lead to occurrence of resistant strains against important antibiotics—relevant in the treatment of *P. aeruginosa* infections—such as β-lactams, quinolones, aminoglycosides, and colistin. The occurrence of a specific resistotype of *P. aeruginosa*, namely the emergence of carbapenem-resistant but cephalosporin-susceptible (Car-R/Ceph-S) strains, has received substantial attention from clinical microbiologists and infection control specialists; nevertheless, the available literature on this topic is still scarce. The aim of this present review paper is to provide a concise summary on the adaptability, virulence, and antibiotic resistance of *P. aeruginosa* to a readership of basic scientists and clinicians.

## 1. Introduction, Taxonomy, and Microbiology of *Pseudomonas aeruginosa*

### 1.1. General Concepts

Non-fermenting Gram-negative bacteria (NFGNB) are a taxonomically heterogenous and populous group of *Proteobacteria* [1]. While the phenotypic characteristics of these microorganisms may be quite distinct, most of the NFGNBs are obligate aerobic, motile (presenting with polar or peritrichous flagella, with the exception of *Burkholderia mallei*, which is non-motile), and oxidase-positive (i.e., they use simple carbohydrates, e.g., glucose in an oxidative fashion) rods. In addition, they are all similar in that they are unable to ferment sugars (hence the name of the group) to generate energy for their vital biological functions [2,3]. NFGNB include some commonly isolated genera, such as *Pseudomonas*, *Acinetobacter*, the *Burkholderia cepacia* complex (BCC), and *Stenotrophomonas* (*Xanthomonas*) *maltophilia*; nevertheless, other less frequently isolated genera should also be taken into consideration, e.g., *Achromobacter*, *Alcaligenes*, *Brevimundas*, *Elisabethkingia*, *Flavobacterium*, and *Ralstonia* [4,5,6]. 

### 1.2. Taxonomy and Phenotypic Characteristics of P. aeruginosa

Based on phenotypic characteristics, Gilardi has classified NFGNB into seven main groups, while Palleroni has differentiated five distinct homologous rRNA groups (namely, I.: *Pseudomonas*, II.: *Burkholderia*, III.: *Comamonas*, IV.: *Brevimundas*, and V.: *Stenotrophomonas*) based on rRNA-DNA homology [7,8]. *P. aeruginosa* was first isolated from green pus by Gessard in 1882, while the genus *Pseudomonas* was first described by Migula in 1894, with *P. aeruginosa* being the species type of the genus [9]. Members of the *Pseudomonaceae* family are ubiquitous in nature (soil, plants, and aquatic environments, while birds and smaller mammals have also been described as reservoirs) [10,11,12]. *P. aeruginosa* is the most common cause of infections (both within the genus and among NFGNB) in humans and warm-blooded animals (e.g., urinary tract infections, mastitis, and endometritis in livestock and companion animals) [13]. Other members of the genus are relevant as fish pathogens, causing hemorrhagic septicemia and ulcerative syndrome [14,15]. *P. fluorescens* and *P. putida* have been described as a cause for deterioration of refrigerated food, and as contaminants in blood transfusion and infusion preparations. *P. stutzeri*, *P. mendocina*, *P. fulva*, and *P. monteilii* are rarely pathogenic in humans (described in patients with end-stage disease and in septicemia), while *P. baetica*, *P. syringae*, *P. plecoglossicida*, and *P. viridiflava* are important plant pathogens [16,17,18]. 

**Figure 1:**
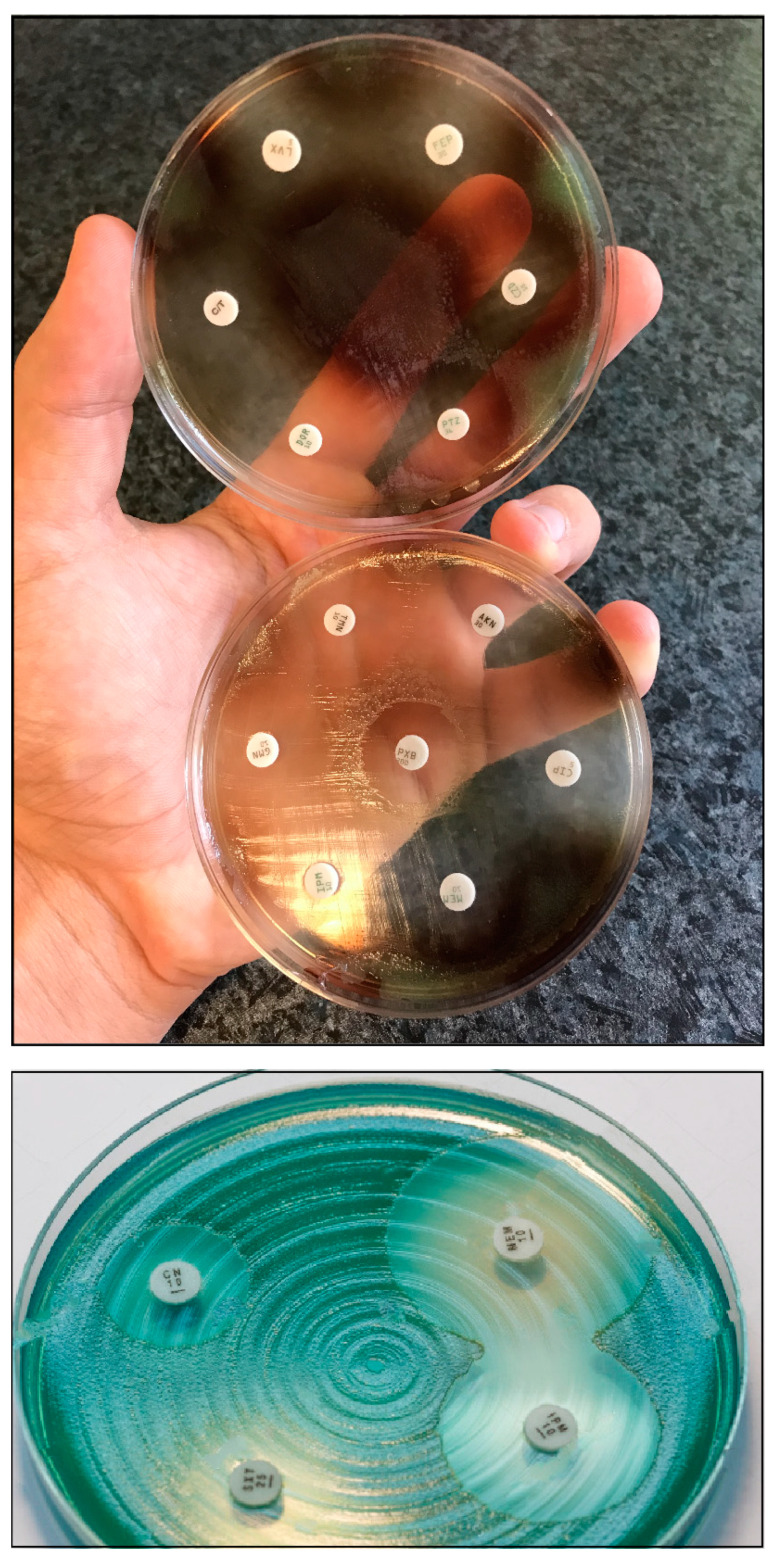
*P. aeruginosa* antimicrobial susceptibility testing using disk diffusion on Mueller-Hinton agar plates. The isolate on the upper portion of the figure produces pyorubin and pyomelanin, while the isolate on the lower portion of the figure produces pyocyanin.

*P. aeruginosa* is a non-fastidious microorganism that does not require special cultivation conditions. It grows well on most non-selective (Mueller-Hinton, Nutrient agar, Luria-Bertani, blood agar, etc.) media, although there are some media which are used specifically for the purpose of selective propagation of *Pseudomonas* (e.g., cetrimide agar, King-A, and King-B media). While the microorganism grows best at 37 °C, pseudomonads can also survive in a wide temperature range (4–40 °C) [19,20]. Among the phenotypic characteristics of *P. aeruginosa*, the characteristic odor (described as flower-like, “grape juice”, or “fresh tortilla”), β-haemolysis (on blood agar), and color of the colonies (in appropriate culture media) allows for their quick organoleptic identification [21]. *P. aeruginosa* and other members of the genus are known to produce various pigments, including pyoverdine/fluoresceine (a fluorescent green-yellow water-soluble pigment, produced by 70–80% of isolates, which also acts as a siderophore in low-iron conditions), pyocyanin (a green-blue lipid-soluble phenazone-derivative pigment, with roles in iron metabolism, in maintaining the redox-equilibrium surrounding the bacteria, and in cell–cell communication), pyorubin (a red-brown water-soluble pigment, produced by 2–3% of isolates, with roles in maintaining the redox-equilibrium), and pyomelanin/alkaptomelanin (a brown-black, water-soluble, and acidic pigment) [22,23,24,25] (Figure 1). It has been shown that high phosphate concentration in the culture media induces pigment production in *Pseudomonas* spp. [26,27].

## 2. Virulence Determinants of *P. aeruginosa* and Modulation of Virulence Factor Expression

### 2.1. Genome of P. aeruginosa

The pathogenicity of *P. aeruginosa* is supported by numerous virulence determinants, some of which are integral parts of their cell structure. On the other hand, many additional virulence factors are synthesized and excreted, depending on the environment surrounding the pathogen [28,29]. One of the most important characteristics of *P. aeruginosa* is its adaptability to diverse natural environments and to harsh (in vivo) conditions, which concurs with high metabolic diversity among species of this genus [30]. The publication of the first sequenced genome of the opportunistic pathogenic strain *P. aeruginosa* PAO1 (isolated from a wound) by Stover et al. in 2000 had paramount importance in shedding light on the physiology and virulence capabilities of this pathogen [31,32]. Since then, the complete genome of many other species of the genus (*P. putida* KT2440, *P. fluorescens* Pf-5, *P. fluorescens* PfO-1, *P. entomophila* L48, and others) have been published [31,32]. In comparison with a common Gram-negative isolate, namely the uropathogenic *Escherichia coli* (UPEC) (with a genome of ≥5 Mb) [33,34], *P. aeruginosa* has a large genome with 5.5–7 Mb, characterized by pronounced genomic plasticity [35,36]. This genetic repertoire includes a conserved core genome of ~4 Mb, while the remaining genetic material comprises of various sets of rare genes and gene islands [37]. The versatility of this pathogen is largely determined by the latter group of genes. 

The *P. aeruginosa* genome resembles a classical “secretor” genome, which includes a large proportion of regulatory genes (i.e., efflux pumps and other transport proteins, motility, chemotaxis), genes controlling metabolic pathways (which allows for adapting to distinct metabolic states), and genes encoding a plethora of virulence factors and antibiotic resistance determinants [38,39]. For example, cystic fibrosis—the defect of the cystic fibrosis (CF) transmembrane conductance regulator (CFTR) genes—leads to the accumulation of succinate in the lungs, which favors the colonization and survival of *P. aeruginosa*, as this microorganism can utilize it as a nutrient source [40]. Secreted virulence factors and proteases are some of the hallmarks in *P. aeruginosa* pathogenicity, which take up ~3% of the open reading frames of the *P. aeruginosa* PAO1 genome [31,32,41]. The diversity of the *P. aeruginosa* genome is further enhanced by the introduction of mobile genetic elements via horizontal gene transfer (HGT; such as conjugative transposons, insertion sequences, and genomic islands) [42]. *P. aeruginosa* also has an innate way to increase genetic diversity in hypermutable strains: the DNA-mismatch repair system in these microorganisms consist of a protein trimer (namely the MutS-MutL-UvrD trimer), with the role of maintaining genomic integrity in these species [43,44]. It has been suggested that species with mutations in this repair system result in “hypermutator” strains, where the spontaneous mutation rate is increased 1000×. These isolates are principally seen in the lung of CF patients and they are characterized by phenotypic changes (i.e., the so-called “mucoid” phenotype) and high-level antibiotic resistance [45,46].

### 2.2. Virulence Factors of P. aeruginosa

Similar to other Gram-negative bacteria, lipopolysaccharide (LPS, or endotoxin), Type IV pili and flagella, adhesins, and lectins are all integral parts of the external cell wall structure of *P. aeruginosa* [47,48]. Based on the O-specific polysaccharide side chain of the LPS, 27 antigen groups may be differentiated, while there is also an opportunity to classify these bacteria based on their flagellar H-antigens [49]. The feature of motility for *P. aeruginosa* is recognized as an advantage, as it is able to move from one niche to another with no difficulty [47,48,49]. Three types of motility, including swarming, swimming, and twitching motility, enable *P. aeruginosa* to be present in a wide range of different habitats with a diversity of environmental factors [50]. Lectins are proteins on the outer membrane of *P. aeruginosa*, which recognize glycosylated carbohydrates on host tissues, aiding the adherence of bacterial cells. For example, LecA (which binds to galactose) and LecB (which binds to fucose) mediate the adherence of this pathogen to epithelial cells in the lung [51,52]. These cell-mediated virulence determinants have important roles in the initial phase of colonization, persistence, and in the establishment of infections in vivo [53]. Nevertheless, the overwhelming majority of virulence determinants associated with *P. aeruginosa* are secreted factors. These may be synthesized and secreted to the vicinity of these bacteria (damaging surrounding tissues and immune cells). In addition, they may be introduced directly into host cells via a type III secretion system (T3SS) [54,55,56]. Secreted virulence factors are relevant in the later stages of the infection and invasion, during which bacterial cells proliferate and subsequent damage occurs in tissue cells at the anatomical site of infection, and the host immune response is dampened [57]. 

These secreted virulence factors in *P. aeruginosa* include: (i) pigments (described previously), siderophores (e.g., achromobactin), and inorganic compounds (e.g., hydrogen cyanide), which have roles in iron scavenging, protection against damage caused by reactive oxygen species (ROS; originating from immune cells), and competition against other bacterial genera. (ii) Exotoxins, including effector cytotoxins such as exotoxin A (ETA), exotoxin S (ExoS; inhibits the function of innate immune cells and neutrophil granulocytes), exotoxin U (ExoU; phospholipase activity, which rapidly leads to cell lysis and has roles in inducing septic shock), and other exotoxins with similar functions (ExoT: inhibits cell division in mammalian cells and affects wound healing processes, ExoY: induces pro-apoptotic processes, and exolysin A (ExlA), which is secreted by a two-partner secretion system (TPS)). (iii) Proteases and other enzymes: lipases, alkaline protease, elastase A (LasA), and B (LasB), heat-stable hemolysin/phospholipase H (PLH), phospholipase C (PLC), and DNase. (iv) Secretion systems: *P. aeruginosa* is known to have 5 types of secretion systems, among which, Types I (T1SS), II (T2SS), and III are involved in the virulence of this pathogen. T1SS and T2SS are relevant in the secretion of various proteases and lipases, ETA, LasA, LasB, and PLH. On the other hand, there are two distinct T3SSs in *P. aeruginosa*: the role of fT3SS is to expel flagellar proteins (to aid in motility, and they may also play a role in biofilm formation), while the iT3SS is a needle-like protein (“injectasome”), which introduces the previously mentioned effector toxins (such as ExoU and ExoS) into the cytoplasm of mammalian cells [42,54,55,56,57,58,59,60,61,62,63]. (v) Biofilm (see Section 2.4). In contrast to cell-mediated virulence factors (which are considered to be constitutive), the production of secreted virulence factors is largely dependent on the environmental factors and the niche surrounding the pathogen.

### 2.3. Typing Methods for the Differentiation of P. aeruginosa Clones, Global Dissemination

Many methods (with various costs, labor-intensity, and discriminatory power) have been proposed for the assessment of genetic similarity in *P. aeruginosa*, which are just as important for local infection control interventions and outbreak control as they are relevant in the assessment of successful national or global clones by public health microbiology [64,65]. These methods include serotyping, phage typing (based on the differential sensitivity to these isolates to standardized bacteriophages), pyocin typing, pulse-field gel electrophoresis (PFGE), field-inversion gel electrophoresis (FIGE), random amplified polymorphic DNA polymerase chain reaction (RAPD-PCR), oligonucleotide microarrays, multi-locus sequence typing (MLST), and whole-genome sequencing (WGS) [66,67,68]. While the latter three methods are relevant in the identification of internationally successful *P. aeruginosa* clones, the other listed typing methods are used in the assessment of local outbreaks. Currently, three major international multidrug-resistant (MDR) clones have been identified, which have shown the most successful spread around the globe, namely the ST111, ST175, and ST235 clones [69,70]. ST111 (characterized by serotype O12) and ST235 (characterized by serotype O11) have been described on almost every continent of the world, while ST175 (characterized by serotype O4) has only been detected in European countries [69,70].

ST235 clones are known as highly virulent—owing to the presence of ExoU in these strains—and these isolates are MDR; thus, the therapy of these infections is also considerably more difficult. Generally, it may be said that the continuous expression of resistance-determinants hinders the virulence of the microorganism; however, the fitness burden associated with maintaining the MDR-phenotype was observed to be lower in case of the ST235 clones [71]. Based on WGS analysis, *P. aeruginosa* isolates of clinical and environmental origin may be grouped into three distinct resistotypes, namely PAO1, PA14, and PA7. PAO1 and PA14 are characterized by possessing the T3SS secretion system and the corresponding effector toxins (ExoS but not ExoU in the case of PAO1, and ExoU but not ExoS in the case of PA14). On the other hand, PA7 does not have the T3SS; instead, they utilize the TPS, by which they secrete the ExlA exolysin to damage surrounding tissue cells [72]. Some reports suggest that there may be an association between virulence and antibiotic resistance in *P. aeruginosa* isolates, as the carriage of the exoU genes was shown to correlate with resistance to aminoglycosides and fluoroquinolones. A possible explanation was that the genomic island carrying exoU may also contain resistance-determinant genes [73,74].

### 2.4. Biofilm Formation

Without a doubt, one of the most important virulence determinants in the pathogenesis of *P. aeruginosa* infections is the production of a biofilm. The biofilm allows for the adherence of these pathogens on various surfaces, provides protection from harsh environmental conditions (e.g., sheer forces, drying), and from the immune system of the host (e.g., natural killer cells, phagocytes, complement, ROS-mediated damage) [75,76,77]. Biofilms have heterogenous compositions, consisting of aggregates of sessile bacterial communities (based on their composition, this may be monospecies or multispecies biofilm), exopolysaccharides (EPS; e.g., alginate, cellulose, dextran, rhamnolipids), environmental DNA (eDNA), carbohydrates, proteins, surfactants, lipids, various ions, and water [78,79]. The biofilm mode of growth was first described in the 1930s, while the true relevance of biofilm-embedded bacteria in infectious processes has been understood only in recent decades [80,81]. Bacterial cells usually attach to hydrophobic and/or coarse surfaces with the aid of their cell-mediated virulence determinants (e.g., pili, fimbriae, surface antigens), which is followed by the production of the protective EPS and other components [82]. Biofilms allow *P. aeruginosa* to persist in the external environment (in water pipes and tanks, sinks, on hospital tiles, on medical equipment, such as mechanical ventilators and respiratory tubing, humidifiers, dialysis equipment and catheters, endoscopes and implanted medical devices, in medical preparations, such as irrigation solutions, dialysis fluid, contact lens fluid, antiseptic solution, cremes) and in vivo [75,76,77,78,79,83,84]. 

Biofilm formation is a critical attribute of *P. aeruginosa* in being a successful nosocomial pathogen and it is also an important hallmark of chronic bacterial persistence. This may be observed in dental caries on the tooth surfaces [85,86], in skin and soft tissue infections [52], in infections of the middle ear [87], catheter-associated infections [19], pneumonia, and in the lungs of CF patients [88]. In the latter case, *P. aeruginosa* is able to survive and avoid clearance (withstanding the immune response and the subsequent administration of antimicrobials) in the respiratory and conductive zone of the lungs [89,90]. For example, alginate and other polysaccharides produced by the mucoid variants are effective in scavenging ROS, protecting bacterial cells [91]. Other than the protection against immune cells, the biofilm provides a safe haven for microorganisms against antibiotics in vivo, contributing to the MDR phenotype. It has been noted by several publications that the minimum inhibitory concentrations (MICs) of bacteria inside the biofilm may be 10–10,000 times higher, compared to planktonic cells [75,76,77,78,79,83,84,88,89,90]. On one hand, the secreted extracellular matrix significantly hinders the diffusion of the antibiotic molecules to effectively reach the bacterial targets (pharmacokinetic barrier); in addition to this, bacteria residing in the deeper layers of the biofilm will adapt to a differentiated metabolic state [75,76,77,78,79,83,84,88,89,90]. It must be noted that the inhibition of bacterial growth is mechanistically distinct from bacterial killing, and antimicrobials (even in effective doses) may not kill cells inside a biofilm. Due to the high bacterial density, low oxygen tension, and lack of nutrients, bacteria become dormant and utilize alternative metabolic pathways [91].

In addition to lacking cell motility, these “persister” cells (also termed small-colony variants (SCVs)) correspond to a transient phenotypic variant of bacteria, which are not genetically resistant to antibiotics, but under the abovementioned conditions, they can withstand very high concentrations of these drugs (pharmacodynamic barrier) [92,93,94,95]. In essence, persisters (corresponding to 1–2% of the bacterial population) opt not to proliferate during exposure to antibiotics, but they resume replication if the stressors are removed from the environment [92,93,94,95]. Persisters may also be important in the recurrence and chronicity of *P. aeruginosa* infections. Although there is scarce knowledge on the mechanisms leading to dormancy/persister formation, it has been suggested that the secretion systems may have a role [96]. The therapy of biofilm infections is an important challenge, as there is currently no targeted therapy available to completely eradicate biofilms in vivo. Nevertheless, several compounds (e.g., polyvalent anions, DNases like dornase-α and alginate lyase) may be useful in the reduction of mucus density [97]. On the other hand (although the evidence on this topic is still controversial), some experiments have shown that sub-MIC concentrations of some antibiotics (mainly β-lactams, including ceftazidime, cefepime, imipenem, and meropenem) may have the opposite effect, inducing biofilm production [98,99,100]. *P. aeruginosa* also displays the ability to tolerate biocides (e.g., antiseptics and disinfectants) like chlorhexidine or triclosan, mediated by the *fabV* gene, coding for a triclosan-resistant enoyl-acyl-carrier protein. Lack of susceptibility to biocides further hinders successful elimination of *P. aeruginosa* from hospital environments [101,102]. 

### 2.5. Quorum Sensing (QS)-Mediated Control of Virulence Factor Expression in P. aeruginosa

**Figure 2:**
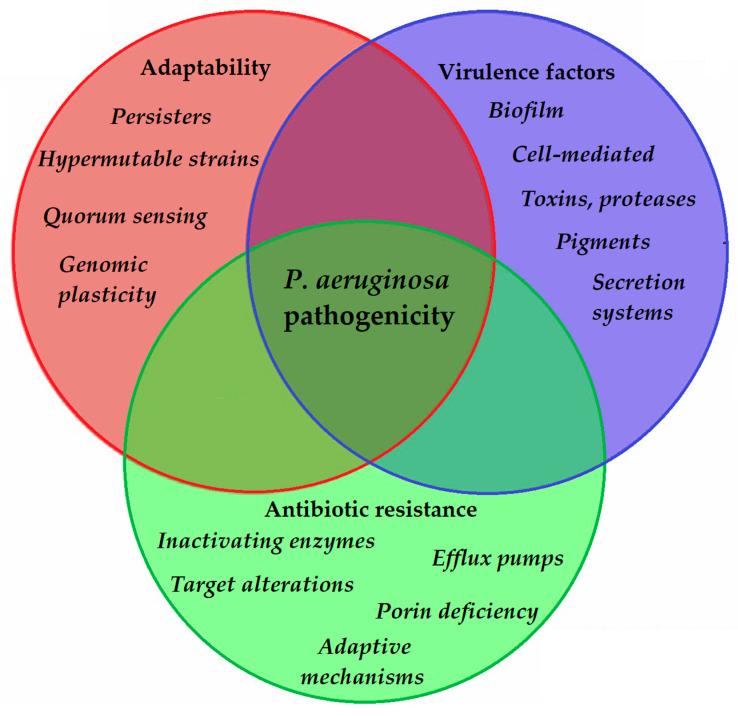
Main components of *P. aeruginosa* pathogenicity.

To allow for the continuous adaptation of *P. aeruginosa* to different environmental niches and to the different stages of infection, the secretion of the abovementioned virulence factors needs to be tightly regulated. One of the most important regulators in *P. aeruginosa* is by its quorum sensing (QS) systems [103]. QS corresponds to the “social behavior” of bacteria, during which small signal molecules (termed autoinducers) are used to influence gene expression in bacterial cells, in a cell-to-cell and density-dependent manner [104,105]. If the density of the bacterial population (hence, the concentration of these signal molecules) reaches a certain threshold, changes occur in bacterial physiology to aid collective behaviors or to help microorganisms to outcompete other microorganisms in the ecological niche (e.g., by secreting virulence factors or antibacterial compounds) [106,107]. Four interconnected systems, namely the *iqs*, *las*, *pqs*, and *rhl* pathways, compose the QS-regulatory network of *Pseudomonas* species. In this network, various autoinducers (such as acyl-homoserine lactones (acyl-HSLs), like butanoyl homoserine lactone (C4 HSL) and 3-oxodecanoyl homoserine lactone (C12 HSL)), the *B. cepacia* complex fatty acid molecule named diffusible signal factor (BDSF), oligopeptide-type autoinducers (like autoinducer-2 (AI-2)), the *Pseudomonas* quinolone signal molecule (PQS), and integrated QS signal molecule (IQS)) are utilized. The detailed description of these signal molecules is outside of the scope of this review (for details, see References [103,104,105,106,107,108,109,110,111,112,113]). Additionally, these autoinducer molecules are capable of dampening the innate immune response and inducing cytokines and chemokines [114]. As the production of biofilm and the secretion of other virulence factors are all governed by the complex QS system of *P. aeruginosa*, they have significant influence on the virulence of these bacteria. QS mediates the expression of its pigments, alkaline protease, hemolysin, elastase, lectins, the effector exotoxins, exotoxin A, swimming and twitching motilities, the activity of the T1SS and T2SS (the activity of T3SS is influenced by QS to a lesser extent), production of biofilm, and hydrogen cyanide, among others [115,116]. QS is also an important mediator of the reciprocity between bacterial virulence, antibiotic resistance, and microbial fitness [117]. The complexity of *P. aeruginosa* pathogenicity is represented in Figure 2.

Nonetheless, it is well-known that the upkeep of many resistance determinants and virulence factors may bear high fitness costs, leading to more susceptible strains outcompeting MDR ones [103,104,105,106,107,108,109,110,111,112,113,116,117]. Conversely, therapeutic and sub-inhibitory doses of antibiotics (e.g., ceftazidime, cefepime, imipenem, fluoroquinolones, doxycycline) may eradicate various QS signal molecules or inhibit their binding to the relevant receptors; thus, suppressing their virulence [118,119]. In addition to QS, biofilm production is also mediated by various two-component regulatory systems (GacS/GacA, RetS/LadS) and cyclic diguanylate (cyclic-di-GMP; a cyclic dimeric guanosine monophosphate), with the signal molecule having critical roles in the secretion of EPS [120]. If these bacteria adhere to any in vivo or inanimate surfaces, the concentration of cyclic-di-GMP increases, leading to the expression of “static” determinants, such as adhesive pili (securing attachment to the surface) and the subsequent production of biofilm. At the same time, increased cyclic-di-GMP also results in the repression of the synthesis and function of flagella (“motility” determinants) [120,121]. This leads to the thickening of the initial biofilm, corresponding to protection against immune cells and antimicrobials. 

### 2.6. Clinical Relevance of P. aeruginosa

As described previously, *P. aeruginosa* has the means to migrate, evade host immune responses and noxius antimicrobial agents, produce toxins and exoenzymes to damage the host cells, and to successfully adapt to any environment [122]. In the consideration of global epidemiological features of NFGNB, *P. aeruginosa* is the most frequent cause of infections [123,124]. Even though pseudomonads do possess various virulence factors, when compared to members of the Enterobacterales order or to other bacteria more commonly seen as pathogens (e.g., *Staphylococcus aureus*, *Streptococcus pyogenes*), they are not considered as highly pathogenic. Nevertheless, they may still be responsible for a wide range of disease manifestations and these pathologies often manifest as chronic, hard-to-eradicate infections [125,126]. Multisite infections are also common for *P. aeruginosa*. Persons affected are most commonly immunocompromised patients (affected by other diseases or underlying conditions, see Table 1), but its pathogenic role should not be disregarded in immunocompetent patients [125,126,127,128,129,130]. *P. aeruginosa* is mainly considered an opportunistic, nosocomial Gram-negative pathogen (responsible for 13–19% of hospital-acquired infections in the US), which is commonly found in intensive care units (ICUs) and surgical theaters, where the extensive use of antimicrobials has allowed for the selection of these microorganisms [131]. Practically all healthcare institutions have reported *P. aeruginosa* outbreaks and intrahospital infections, as these bacteria have the ability to persist on a plethora of inanimate surfaces and to spread via an aerosol [132]. Under normal circumstances, *P. aeruginosa* can only transiently colonize the intestinal tract (although this rate may increase if the patient is immunocompromised). Nevertheless, 8–20% of nosocomial infections and outbreaks are associated with colonized individuals [125]. To avoid nosocomial outbreaks, the strict adherence to infection control protocols, environmental cleaning plans, and hand hygiene practices are of critical importance, in addition to the identification and elimination of possible reservoirs of infection [125].

Possible clinical manifestations include pneumonia (mainly ventilator-associated (VAP; 10–30%), while the community-acquired form is far less common (CAP; 1–3%)), skin and soft tissue infections associated with burns and surgeries (8–10%), “hot tub” folliculitis, “swimmer’s ear” otitis externa, eye infections (keratitis), urinary tract infections (UTI; 813.8615%), endocarditis, and bacteremia/sepsis (central-line-associated or often secondary to pneumonia) [125,126,127,128,129,130,133]. Among bacterial pathogens responsible for contact lens-associated keratitis, *P. aeruginosa* has the worst disease manifestation (i.e., the development of a corneal ulcer, which may occur in 40–60% of cases), leading to poor outcomes, the fulminant destruction of the cornea, and vision loss [73,134,135]. The mortality rate of pseudomonad infections is a big concern among immunocompromised and hospitalized patients, which is around 25–39% for pneumonia and 18–61% for bacteremia, while these rates may be higher (40–70%) in case of MDR isolates [136,137,138]. In some age groups, these infections may have particularly severe manifestations (e.g., nosocomial pneumonia in the elderly, and severe sepsis and meningitis in neonates) [125,126,127,128,129,130,131,132,133,134,135,136,137]. The poor outcomes associated with these infections are corresponding to the severe condition of these patients and to the virulence factors of this pathogen. *P. aeruginosa* is also an important factor in the progression of chronic respiratory disorders, e.g., a systematic review has found increased hospitalization rate, higher exacerbation rate, worse quality of life, and 3-fold increase in the mortality risk in patients with bronchiectasis positive for *P. aeruginosa* in their lungs [139]. *P. aeruginosa* was also found to be induced by cigarette smoke, which has led to the emergence of an *nfxC* drug-resistant phenotype [140]. 

*P. aeruginosa* is the most common and oldest studied pathogen in CF. The first exposure to pseudomonads may be related to a previous viral infection (which eliminates host defenses in the lungs even further) and the bacteria usually originate from some natural source (e.g., aerosols, water, bacterial flora of other individuals) [141]. *P. aeruginosa* strains that settle in the lungs of CF patients are initially not of the mucoid type; however, strains with a highly slimy surface and a mucoid phenotype are isolated 3–6 months later [142]. In CF patients, *P. aeruginosa* may only be eradicated in the early stages of colonization (which provides a ‘window of opportunity’ for early aggressive antibiotic treatment), while during chronic colonization or exacerbations at the later stages of life, the goal is to reduce the number of bacteria [141,142]. Age at first positivity for *P. aeruginosa* was shown to be an important determinant of the disease course in CF-affected individuals. The prevalence of the pathogen between 0 and 5 years of age is 10–30%, while over 25 years of age it is present in >80% of patients and these chronic lung infections are rarely eradicated completely. *P. aeruginosa* is one of the most important factors in fatal pulmonary exacerbations in CF patients [142]. *P. aeruginosa* is also an important pathogen in animal husbandry, making it a considerable cause of economic losses and difficulty keeping up animal stocks for marketing purposes (see Section 1.2) [13,14,15,16,17,18,143].

## 3. Antibiotic Resistance in *P. aeruginosa*: Therapeutic Alternatives

### 3.1. General Concepts Related to MDR Pathogens

As a general rule, the therapy of infections caused by NFGNB present a concerning therapeutic challenge to clinicians both in community and in hospital settings, due to increasing prevalence of resistant isolates, showing resistance to several classes of antibiotics [144]. Nowadays, the emergence of isolates classified as MDR, extensive drug-resistant (XDR), and even pandrug-resistant (PDR, or totally drug-resistant (TDR), defined by the recommendations of the European Society of Clinical Microbiology and Infectious Diseases; ESCMID [145]), is becoming commonplace in clinical practice. The burden of MDR bacteria—and the associated risks for humanity—have been highlighted by many national and international bodies (e.g., the World Health Organization (WHO) [146], the European Center for Disease Control and Prevention (ECDC) [147], the UK National Health Service (NHS) [148], and the US Centers for Disease Control (CDC) [149]), stressing that without the availability of adequate therapy, these infections lead to prolonged hospitalization, decreased quality of life (QoL), sequelae, and excess mortality in the affected patient populations. In many cases, poor clinical outcome is clearly associated with infections caused by MDR pathogens, compared to their susceptible counterparts [150]. The two main driving forces behind the clinical problem of antibiotic resistance is their indiscriminate use in inappropriate indications, in addition to the dwindling interest of pharmaceutical companies to get involved in antimicrobial research [151].

Although the problem of MDR was first observed in hospital-acquired infections, nowadays, it is not uncommon to acquire an infection with a MDR pathogen in the community [152]. The Burden of Antimicrobial Resistance Collaborative Group estimated that in the year 2015 alone, there were over 700,000 MDR infections, 33,110 excess deaths, and around 875,000 disability-adjusted life years (DALY) described in the European Union and European Economic Area [153]. MDR organisms also threaten the progress of the Sustainable Development Goals (SDGs) set by the United Nations for 2030, having the most serious effects in developing countries [154]. *P. aeruginosa*—along with other NFGNB—is a member of the so-called “ESKAPE” pathogens (E: *Enterococcus faecium*, S: *S. aureus* or recently *S. maltophilia*, K: *Klebsiella pneumoniae* or recently C: *Clostridioides difficile*, A: *A. baumannii*, P: *P. aeruginosa*, E: *Enterobacter* spp., or recently *Enterobacteriaceae*): the acronym includes bacteria that are most concerning from a clinical and public health perspective [155]. This has been underlined by the report published by the WHO, in which carbapenem-resistant *P. aeruginosa*, carbapenem-resistant *A. baumannii* complex, and carbapenem-resistant or ESBL-producing members of Enterobacterales were all designated as critical priority pathogens [156]. The most worrisome reports in the international literature have emerged regarding MDR *Acinetobacter* spp.; nevertheless, due to its much higher incidence, the relevance of *P. aeruginosa* is more pronounced [157]. Recently, a PDR strain of *Pseudomonas* sp. strain has been described, which was capable of using ampicillin as a sole carbon source [158]. 

### 3.2. Intrinsic Resistance and Main Therapeutic Alternatives in P. aeruginosa Infections

The therapy of *Pseudomonas* infections heavily relies on a limited number of antibiotics, and some recently marketed novel agents are relevant in case of extensive resistance (Table 2) [159,160]. The modes of antibiotic resistance in *P. aeruginosa* may be classified into three categories, including (i) adaptive (i.e., biofilm formation, dormant forms), (ii) intrinsic (see Table 2), and (iii) acquired resistance mechanisms (mutation or acquisition of integrons, plasmids, prophages, and transposons by the means of HGT), resulting in a rich and diverse resistome [161]. The occurrence of mutations may lead to antibiotic uptake reduction, antibiotic target modifications, and over-expression of both efflux pumps system and antibiotic inactivating enzymes [162,163,164]. In essence—apart from the fluoroquinolones (who present with excellent oral bioavailability)—all of the present therapeutic alternatives for *P. aeruginosa* need to be administered parenterally [165]. Clinically, infections caused by *Pseudomonas* spp. are most frequently treated with β-lactam antibiotics: these drugs should be considered as the backbone of anti-infective therapy, especially in case of special patient groups (i.e., infants, young children, pregnant women, and in the elderly). In these patients, the use of other ancillary drugs is contraindicated, due to their adverse events (neurotoxicity and nephrotoxicity for aminoglycosides and colistin, and tendonitis, tendon rupture, photosensitivity, and hepatotoxicity for fluoroquinolones) or due to their teratogenicity [166,167]. If resistance levels become more and more advanced (or in the case of hypersensitivity to β-lactams), last-resort antibiotic regimens may be needed, which correspond to more severe adverse events and decreased quality of life (QoL) in the affected patients [168]. Nephrotoxicity is a critical concern in transplant patients, who receive several other medications affecting the kidneys [136]. In fact, a novel classification method for bacterial resistance termed difficult-to-treat resistance (DTR) takes into consideration the clinical usefulness and the risk/benefit ratio of antibiotics in the treatment of Gram-negative infections. Based on this criterion, *Pseudomonas* isolates resistant to broad-spectrum cephalosporins, carbapenems, and quinolones are termed DTR [169,170]. The risk factors for the acquisition of MDR *P. aeruginosa* include the admission to an intensive care unit (ICU), prior hospital stay, and previous use of various antibiotic groups (quinolones, cephalosporins, carbapenems) [171,172]. Interestingly, proteases and elastases (two important virulence factors for the development of serious, invasive disease) are also the most common in isolates originating from the ICU [173].

Currently, antibiotic dosing strategies (as they are primarily dosed aiming to cure) facilitate the emergence of resistant mutant subpopulations in bacteria; therefore, a change in clinical approach needs to occur to shift the endpoint of therapy towards killing and the suppression of resistance (which is mechanistically different from inhibition of growth) [174,175]. To achieve this, rapidly lethal agents to these microorganisms need to be applied in sufficently large doses, which also simultaenously results in a clinical cure [174,175].

### 3.3. Main Mechanisms of Resistance in P. aeruginosa to Antibiotics Other Than β-Lactams

The Gram-negative cell wall is a complex construct of bacterial anatomy (including the asymmetric bilayer of phospholipid, penicillin-binding proteins (PBPs), porins (or outer membrane proteins (OMPs)), and other types of protein channels, LPS, and various efflux pumps), which acts as a selective barrier to be penetrated or bypassed for antibiotic molecules to be able to exert their pharmacological activities on their molecular targets [176]. The permeability of the *P. aeruginosa* outer membrane is restricted (10–150× lower than that of *E. coli*), leading to the intrinsic non-susceptibility to many previously listed agents (coupled with their efflux). In addition, any changes to the constituents of this cell wall structure will unavoidably affect the susceptibilities of antibiotics [177,178]. *P. aeruginosa* porins (β-barrel proteins folding within the outer membrane composed of anti-parallel β-sheets) are classified into non-specific (OprF), specific (OprB, OprD or the D2 porin, OprE, OprO, OprP), gated (OprC, OprH), and efflux (OprM, OprN, OprJ) porins [179]. Among different porins of *P. aeruginosa*, OprF is the most common non-lipoprotein within the outer membrane (the *E. coli* homolog porin is OmpA), which is involved in securing the integrity of the outer membrane, QS, biofilm formation, bacterial adhesion, and acute and chronic infections [179,180]. β-lactam antibiotics and fluoroquinolones enter bacterial cells through the abovementioned porin channels, aminoglycosides are taken up by a two-step process, involving the presence of oxygen- or nitrogen-dependent electron transport chains, while colistin facilitates its own uptake by interacting with the Gram-negative LPS [176,177,178,179,180]. The absence of oxygen (e.g., in the depths of a biofilm or in anaerobic bacteria) or the functional deficiency of ATPases may lead to resistance against aminoglycosides [181]. The most important porin in the context of antibiotic uptake is the OprD porin, which is a 54 kDa protein. Loss of the OprD porin (usually mediated by the inactivation of the OprD gene through deletions, mutations, or insertions) has been reported as one of the principal mechanisms of carbapenem resistance in *Pseudomonas* [182]; in addition, imipenem selects for porin-deficient mutants in 1 out of 5 patients treated with this drug [183]. On the other hand, the overproduction of the OprH porin (which is the smallest porin of *P. aeruginosa*), coupled with cation-starvation, has been described in isolates with increased MICs to polymyxins and aminoglycosides [184]. 

The over-expression of efflux pumps is a well-known contributor to the MDR phenotype, as it may affect many different groups of antibiotics at once [185]. Based on their protein structures, these efflux systems may be classified into five superfamilies, including the ATP-binding cassette (ABC) superfamily, multidrug and toxic compound extrusion (MATE) family, major facilitator superfamily (MFS), resistance-nodulation-division (RND) family, and small multidrug resistance (SMR) family. On the other hand, they may also be divided into pumps dependent of hydrolyzing ATP to extrude relevant compounds or by utilizing the proton motive force (PMF) [186,187]. From the standpoint of antibiotic resistance in pseudomonads, members of the RND superfamily have the most importance: so far, twelve RND pumps have been described, out of which, the four over-expression ones (mediated by mutations in the *nalB*, *nfxB*, and *nfxC* genes) are significant contributors with varying substrate profiles (MexAB-OprM: β-lactams and quinolones, MexCD-OprJ: β-lactams, MexEF-OprN: quinolones, and MexXY-OprM: aminoglycosides) [186]. Efflux pump-mediated resistance usually confers low-level resistance to relevant antibiotics, which will in turn lead to clinically relevant resistance, if combined with some other resistance mechanism [176,185,186,187,188]. It must be noted that the abovementioned resistance mechanisms may differently affect the in vitro susceptibility of *P. aeruginosa* to individual antibiotics (even in the pharmacological group): in clinical microbiological laboratories, it is often observed that some isolates are resistant to meropenem, but not imipenem, or resistant to amikacin, but not tobramycin [189]. Other than efflux pump overexpression, mutations in the target genes coding for DNA gyrase (*gyrA*, *gyrB*) and topoisomerase IV (*parC*, *parE*) in the QRDR (quinolone resistance-determining region) is the most important mechanism of resistance against fluoroquinolones, leading to decreased binding affinity of these proteins to the drugs [190]. 

In addition to the previously described resistance mechanisms (efflux, decreased membrane permeability), the resistance against aminoglycosides may be mediated by target modification in the 30S ribosome, or by the production of aminoglycoside-modifying enzymes (AMEs; e.g., nucleotidyltransferases, acetyltransferases, phosphotransferases), and 16S rRNA methyltransferases. These enzymes act via irreversibly modifying the chemical structure of these drugs (most commonly the amino and glycoside groups) [191,192]. These modifying enzymes are commonly acquired through HGT. Colistin is currently considered a last-resort, life-saving antibiotic for XDR Gram-negative infections, and due to the developments of antimicrobial resistance, the use of this agent is on the rise (despite the disadvantageous side-effect profile and difficult dosing of the drug) [193]. Before the early 2010s, colistin resistance was only described via mutations in chromosomal genes (i.e., *ccrB*, *mgrB*, *pmrAB*, *phoPQ*), which were transferred vertically [194]. Consequently, plasmid-mediated colistin resistance was detected in 2015, where a plasmid-borne transmissible mobile colistin resistance (mcr-1) plasmid was found [195]. This proved to be a critical concern, as the widespread dissemination of colistin resistance through mobile genetic elements was now a possibility. To date, ten different *mcr* genes (*mcr*-1 to *mcr*-10) have been described with many variants, from all countries apart from Antarctica [196]. This resistance is mediated by the arnBCADTEF operon, and results in the addition of 4-amino-4-deoxy-L-arabinose and phosphoethanolamine to the lipid A component of the Gram-negative LPS; this, in turn, will lead to the capacity of colistin to interact with the LPS, blocking the self-facilitation of uptake [197]. In case of low concentration of bivalent cations (Mg^2+^ and Ca^2+^), a two-component regulatory system (PhoPQ and PmrAB) is activated, which also leads to colistin resistance [198].

### 3.4. Main Mechanisms of β-Lactam-Resistance in P. aeruginosa

As previously mentioned, β-lactams are the most commonly used therapeutic choices for *Pseudomonas* infections, with carbapenems often being the last safe therapeutic alternatives in many MDR infections [199]. This is especially true since the global emergence and spread of extended-spectrum β-lactamase-producing (ESBL) gut bacteria [200]. Due to the extensive use of these life-saving drugs, the emergence of carbapenem resistance in NFGNB and in gut bacteria (e.g., *E. coli*, *Klebsiella*, and *Enterobacter* species) is steadily increasing [201]. Among NFGNB, *S. maltophilia* has intrinsic carbapenem resistance (due to two chromosomally encoded metallo-β-lactamases), while in case of *Acinetobacter* and *Pseudomonas*, acquired mechanisms are more common [202,203]. Based on the results of a recent meta-analysis, the acquisition of carbapenem-resistant *P. aeruginosa* was associated with not only the prior use of carbapenems, but the history of receiving piperacillin-tazobactam and vancomycin [173]. β-lactam-resistance may be mediated by a plethora of resistance mechanisms, including mutations (downregulation or absence) in porin channels, efflux pump overexpression, and changes in the PBPs, with the most common being the expression of various β-lactamases (while many References stress that carbapenem resistance is usually caused by a combination of different factors) [204,205]. Nevertheless, β-lactamases show a very diverse spectra of activity and their potency is also influenced by their phenotypic expression levels in the isolates [206]. 

Specific modifications in the PPBs may lead to decreased susceptibility to β-lactams, although this mechanism is less common compared to Gram-positive bacteria: alterations in PBP1 usually affect cephalosporins, PBP2 is important for both cephalosporins and carbapenems, changes in PBP3 affect the binding affinity of imipenem, while PBP4 is relevant to the binding of imipenem and meropenem [207]. *Pseudomonas* species are characterized by a chromosomally encoded ampicillin C-type (AmpC) β-lactamase, which is expressed at low levels under normal circumstances; however, the administration of many antibiotics may lead to this enzyme being induced and stably de-repressed (strong inducers include ceftazidime, carbapenem, and clavulanic acid (e.g., in ticarcillin-clavulanate)), leading to high-level resistance to cephalosporins with anti-pseudomonal activity, while sparing carbapenems [208,209]. In addition, loss-of-function mutations in the *ampD* gene (which encodes for the repressor compound of AmpC-expression) also result in hyperproduction of this β-lactamase [210]. For this reason, *Pseudomonas* species are members of the “SPACE” organisms (*Serratia*, *Pseudomonas*, *Acinetobacter*, *Citrobacter* and *Enterobacter*), characterized by the presence of inducible AmpC-based resistance. AmpC activity is not inhibited by first-generation β-lactamase-inhibitors, such as clavulanic acid, sulbactam, or tazobactam [211]. Other than AmpC, *Pseudomonas* spp. may carry a plethora of narrow-spectrum or broad-spectrum β-lactamases. Among β-lactamases, plasmid-borne carbapenemases (either serine- or metallo-β-lactamases) possess the broadest spectrum of substrates (including most penicillins, cephalosporins, and carbapenems); thus, they eliminate these drugs as possible therapeutic options [212,213]. While Ambler Class A carbapenemases, such as KPC (*K. pneumoniae* carbapenemase) and GES (Guiana Extended spectrum, especially GES-2), and Class D enzymes like members of the OXA-48-like family (Oxacillinase) may also be detected in carbapenem-resistant *Pseudomonas*, the principal type of carbapenemases is this genus are metallo-β-lactamases (MBLs) [214,215,216]. In fact, the first transferable carbapenem resistance determinant via MBLs was reported in a *P. aeruginosa* isolate in Japan (1991; Imipenemase 1, IMP-1) [217]. The discovery of IMP-1 was followed by the description of VIM-1 (1997; Verona Imipenemase 1, VIM-1) in a *P. aeruginosa* isolate [218]; in both cases, genes coding for the MBL were found on a gene cassette inserted into a class 1 integron. Since then, a variety of novel MBLs have been described in Gram-negative bacteria, including AIM (Australian Imipenemase), DIM (Dutch Imipenemase), GIM (German Imipenemase), KHM (Kyorin University Hospital), NDM (New Delhi MBL), SIM (Seul Imipenemase), SPM (Sao Paulo MBL), and TMB (Tripoli MBL) [219,220]. In laboratory conditions, carbapenemases may be differentiated through the inhibition of various molecules (e.g., ethylenediaminetetraacetic acid for MBLs, boronic acid for serine-type enzymes), but it is more difficult to carry out due to the low outer membrane permeability of *P. aeruginosa*, which may significantly alter the resistance phenotype [221,222].

Carbapenem-resistant *Pseudomonas* is a major concern in low- and middle-income countries. Infections and colonization with carbapenem-resistant Gram-negative bacteria have been associated with increased economic costs, longer hospital stays, and poorer clinical outcomes compared to their carbapenem-susceptible counterparts [223]. Patients with bacteremia caused by carbapenem-resistant *Pseudomonas* had 3-times higher odds of mortality compared to the infections by carbapenem-susceptible species [224]. Based on the international literature, carbapenem resistance rates in *Pseudomonas* range between 10% and 50%, with pronounced geographical differences [225]. The CDC has reported that carbapenem resistance rates were up to 12% in some parts of the US [226]. The population weighted mean proportion of carbapenem-resistant *P. aeruginosa* was 17.2% in 2018, while this ratio was 30.7% in China in the same year [144]. In Europe, Greece has a very high prevalence of carbapenem resistance in *Pseudomonas* (40.4% in 2015, based on the data of the European Antimicrobial Resistance Surveillance Network), which corresponds to the extensive use of these drugs [227]. Earlier reports (before 2000) highlighted the critical role of OprD inactivation and efflux pumps in carbapenem resistance, while newer reports show that carbapenemases are increasingly displaying a critical role [228]. The highly successful clone ST235 is one of the major carbapenem-resistant clones, which have spread worldwide. This clone usually carries metallo-β-lactamases, including IMP, NDM, and VIM enzymes [38,70,71].

### 3.5. Carbapenem-Resistant but Cephalosporin-Susceptible P. aeruginosa (Car-R Ceph-S)

Resistotyping is a relatively old method of differentiation among bacterial strains, which has garnered new-found interest in the era of extensive resistance in bacteria [229]. The principle of resistotyping includes testing bacterial strains against arbitrarily selected chemical agents (i.e., clinically used antibiotics in microbiology laboratories) to differentiate them by the presence/absence of resistance to selected chemical substrates, whereby a resistance pattern—characteristic for the geographical region for those strains—is generated [229]. The occurrence of a specific resistotype of *P. aeruginosa*, namely the emergence of cephalosporin-susceptible (Car-R/Ceph-S) strains among carbapenem-resistant isolates, has received substantial attention from clinical microbiologists and infection control specialists; however, the available literature on this topic is still scarce. In the following section, our aim was to collect and summarize the currently available data at this time regarding this uncommon resistotype, including epidemiological papers and studies on their phenotypic and genotypic characterization. Most of the reports originate from the Far East, while some publications are also available from the European Continent and the Middle East. 

In a study spanning a three-year-long period, Ferreiro et al. recorded sixty-two (*n* = 62) Car-R/Ceph-S cases causing nosocomial UTIs: the crude mortality rate in their study was also recorded, corresponding to 17.7% [179]. Shigemura et al. reported seventy-six (*n* = 76) patients (77.6% male patients) with a Car-R/Ceph-S *P. aeruginosa* UTI over four years [230]. Zeng et al. collected Car-R/Ceph-S *P. aeruginosa* in the second half of 2011, and they found *n* = 29 individual isolates. During their study, the isolates were characterized by Western blotting and real-time PCR (RT-PCR) methods. It was shown that the expression of AmpC-type β-lactamases and carbapenemases was not verified in these isolates, while the overwhelming majority showed decreased expression or deletion of the OprD porins as the major determinant of resistance [231]. Li et al. characterized Car-R/Ceph-S *P. aeruginosa* isolated from bacteremia over an 8-year (2010–2017) period: sixty-three isolates were collected, with most isolates presenting with overexpression of efflux pumps and decreased expression of OprD [232]. The findings of Li et al. corresponded to those of Zeng et al., as the production of relevant β-lactamases was not shown in these isolates either. The overall 30-day mortality rate between the years 2010 and 2017 was shown to be 27.0% in the affected patients [231]. During the laboratory study by Wi et al., eighteen (23.3%) ceftazidime-susceptible isolates were found among imipenem-resistant *P. aeruginosa* strains. Overexpression of efflux pump genes (*mexB*, *mexD*, *mexF*, and *mexY*) was seen in 13/18 strains, 2/18 showed AmpC β-lactamase overexpression, while decreased oprD gene expression was identified in 18/18 strains [233]. 

Two-hundred and ninety-three Car-R/Ceph-S *P. aeruginosa* isolates were characterized by Khuntayaporn et al. during a three-year study period. The observed prevalence of resistance determinants was the following: AmpC-type β-lactamase production 3.9%, metallo-β-lactamase (MBL) production 18.5%, efflux pump-overexpression 63.5%, and decreased *oprD* gene expression 93.3%, respectively [234]. Campana et al. reported twenty-five individual Car-R/Ceph-S isolates from various types of clinical samples: >90% had reduced levels of oprD gene expression, while overexpression of efflux pumps or production of major types of β-lactamases (detected by the matrix-assisted laser desorption/ionization time-of-flight mass spectrometry [MALDI-TOF MS] method and RT-PCR) were not shown in these isolates [235]. Zaidenstein et al. reported sixty-seven Car-R/Ceph-S bloodstream infections from monobacterial clinical syndromes over a 5-year period (2010–2014). The authors have noted that in these cases, cephalosporins were considered as relevant therapeutic options [236]. Tsai et al. characterized *n* = 14 *P. aeruginosa* isolates, that were resistant to carbapenems only. Reduced OprD expression was found in 93% (14/15) of the isolates, being the major contributor to selective carbapenem resistance in these bacteria [237]. Pena et al. reported a nosocomial outbreak, caused by a cefepime and carbapenem-resistant, but ceftazidime-susceptible, *P. aeruginosa*, affecting 23 patients in Spain. After molecular characterization of the isolate, it was found that it overexpressed the MexXY-OprM efflux pump and produced an integron-borne PSE-1 β-lactamase [238]. Pournaras et al. collected twelve Car-R/Ceph-S during the year 2003, and none of the strains were positive for carbapenemases; in contrast, 12/12 isolates were positive for *mexB*, 11/12 for *mexY*, while 10/12 were also positive for the overexpression for *mexB* and 5/12 for *mexY*, respectively [239]. 

Khalili et al. identified *n* = 23 Car-R/Ceph-S *P. aeruginosa* isolates (9.5% of tested isolates) over a three-year period (2016–2018). Efflux pump overexpression and β-lactamase-production was assessed phenotypically: 60.9% showed efflux-pump overexpression, while AmpC-hyperproduction was shown in 4.3%. Overexpression of relevant efflux pumps was also verified by RT-PCR in 68.8% of isolates [240]. Lee et al. performed a retrospective, case-control study including patients with Car-R/Ceph-S and pan-susceptible *P. aeruginosa* bacteremia over a 6-year period (2004–2010): *n* = 25 patients were recorded with Car-R/Ceph-S bloodstream infections, their mortality was almost three times higher than the mortality of the control group (72% vs. 26%), and carbapenem-resistance was found as the only independent risk factor for mortality [241]. In a 1-year prospective study on non-fermenting Gram-negatives in Egypt, Wafy et al. found *n* = 29 isolates resistant to carbapenems (meropenem) via phenotypic assays; however, out of these isolates, only *n* = 15 were also resistant to antipseudomonal cephalosporins [242]. Rodulfo et al. aimed to compare the presence of the MDR and XDR phenotype in *Pseudomonas* with the occurrence of various virulence factors. In addition to the presence of Car-R/Ceph-S isolates (reported to be ~13% of the overall pool of isolates), their report highlighted the continuous increase in resistance rates to β-lactam antibiotics and class I integrons between 2009 and 2016 (37.1% vs. 50.0% for piperacillin-tazobactam, 32.3% vs. 50% for ceftazidime, 33.9% vs. 50% for cefepime, 38.7% vs. 65.6% for imipenem, and 37.1% vs. 59.4% for meropenem). They have also found a positive association between the MDR/XDR phenotype and the presence of hemolysin, the *exoU* gene, and integrase I [243]. In the study of Khan et al., the resistance characteristics of keratitis-causing *P. aeruginosa* isolates, originating from Australia and India, were compared [73]. All of the isolates carried a β-lactam resistance gene (*bla*_PAO_), most of the Australian isolates carried *bla*_OXA-396_, while other β-lactam resistance genes were also seen in both Australian and Indian isolates (e.g., *bla*_OXA-486_, *bla*_OXA-488_, *bla*_OXA-396_, and *bla*_OXA-50_), although much less commonly. Resistance rates were 78% for imipenem and 57% for ceftazidime, and 58% for imipenem and 50% for ceftazidime in Indian and Australian isolates, respectively [73].

In one the most recent studies on the topic from Southern Hungary, the occurrence of Car-R/Ceph-S *P. aeruginosa* from UTIs was assessed over a 10-year surveillance period. Overall, fifty-seven such isolates were detected and these bacteria were characterized with phenotypic methods: 4/57 isolates produced carbapenemases, 7/57 isolates showed the overproduction of an AmpC β-lactamase, 31/57 overexpressed efflux pumps, while in the case of 15/57 isolates, no conclusive data could be obtained for the resistance determinants using the phenotypic methods included [244]. Interestingly, the first identified integron-borne MBL was also identified from *P. aeruginosa* in Hungary [245]. Based on the reports described previously, there is a small but relevant number of Car-R *Pseudomonas* isolates (4–20 isolates/year, based on the abovementioned reports), where “older” generation β-lactam antibiotics (i.e., ceftazidime and cefepime) may still be relevant to therapy. This allows for the special, more conscious use of these reserve agents. Choosing these agents instead of colistin offers a possibility for antimicrobial stewardship/colistin-sparing [246]. Whilst with a small margin, the superior susceptibility levels of ceftazidime and cefepime in *P. aeruginosa* were also recently highlighted in the International Network for Optimal Resistance Monitoring (INFORM) Surveillance Program (ceftazidime: 85.1%, cefepime: 86.1%, meropenem: 80.2%) [247]. 

Even though there is growing literature available on the topic, there is no consensus on the most common mechanisms of resistance contributing to the emergence of Car-R/Ceph-S isolates. There are wide-ranging differences in the prevalence of carbapenemases (especially for MBLs), and this may affect the susceptibility to cephalosporins as well [73,230,231,232,233,234,235,236,237,238,239,240,241,242,243,244]. Khalili et al. proposed that the detection of Car-R/Ceph-S isolates is dependent on the absence of carbapenemases [240]. Coupled with the low-to-moderate levels of AmpC production, these isolates may show susceptibility to ceftazidime and cefepime, as demonstrated in these reports. On the other hand, the importance of the OprD porin mutations in the presence of carbapenem resistance has been demonstrated by several studies. In fact, porin alterations are one of the most common resistance mechanisms after repeated exposure to carbapenems [176,177,178,179,180,181,182,183,184,185,186,187,188,189]. The cited literature reports highlight the importance of the continuous surveillance of bacterial pathogens possessing β-lactamases (including phenotypic and molecular methods) to aid antimicrobial stewardship interventions and to help in utilizing the safest and most appropriate therapeutic alternatives available [248].

## 4. Emerging Therapeutic Options for *Pseudomonas* Infections

While there have been advances in the marketing authorization of novel antibiotics and combination therapy in the recent decade, their price and availability may hinder their widespread use in the near future. Additionally, it is questionable how long the new agents can manage the worsening resistance situation [146,157,249,250,251,252,253,254]. With the daunting increase in antimicrobial resistance rates in all types of bacteria, one of the main aims of antimicrobial research is the exploration for new approaches past conventional antibiotics, such as bacteriophages, antimicrobial peptides with diverse structures and mechanisms of action, virulence inhibitors, siderophores, compounds from natural origins (like essential oils), and other adjuvants (e.g., efflux pump inhibitors, monoclonal antibodies) (Table 3) [118,255,256,257,258,259,260,261,262,263,264,265,266,267,268,269,270]. It is possible that in the next couple of decades, these agents will play a major role in the management of serious bacterial infections caused by *P. aeruginosa* and other pathogens of critical importance.

## 5. Concluding Remarks

*P. aeruginosa*, an important member in the group of non-fermenting Gram-negative bacteria, has received substantial attention in recent years, associated with many different disease manifestations, especially in hospitalized and/or immunocompromised patients. The emergence of MDR, XDR, and PDR strains in *P. aeruginosa* is an important public health concern, leading to difficulties in therapeutic choices. β-lactam antibiotics are some of the most important drugs in the therapy of *Pseudomonas* infections, with a special focus on carbapenems, which have been considered as reserve antibiotics since their introduction in the 1980s [271]. Nevertheless, the global increase in the utilization of these drugs (both for infections caused by NFGNB and Enterobacterales) has led to the expansion of carbapenem-resistant strains in all parts of the world [272]. These developments should be considered very alarming. The occurrence of a specific resistotype of *P. aeruginosa*, namely carbapenem-resistant but cephalosporin-susceptible (Car-R/Ceph-S) strains, has received substantial interest, as these rare cases may offer opportunities for using older β-lactam antibiotics, instead of using other last-resort agents with more toxicity, and to limit the development of selection pressure on these bacteria.

## Figures and Tables

**Table 1:** Main risk factors for acquiring *P. aeruginosa* infections [125,126,127,128,129,130].

Hospitalization
Mechanical ventilation
Innate or acquired immunodeficiencies (neutropenia, human immunodeficiency virus [HIV]/acquired immunodeficiency syndrome [AIDS], malignancy)
Invasive medical procedures (surgery, transplantation)
Catheterization (urinary, central venous)
Burns, severe external injuries
Immunosuppressive therapy
Cancer chemotherapy
Radiotherapy
Diseases of the cardiovascular system
Diseases of the respiratory system (e.g., chronic obstructive pulmonary disease, cystic fibrosis)
Diabetes mellitus
Patients living in poor living conditions, malnutrition
Intravenous drug use

**Table 2:** Intrinsic resistance and relevant therapeutic alternatives in *Pseudomonas* infections [159,160,161,162,163,164,165].

Antibiotics to Which *Pseudomonas* Species Are Intrinsically Resistant	Antibiotics Relevant in the Therapy of *Pseudomonas* Infections
Glycopeptides (e.g., vancomycin)	***β-lactam antibiotics:*** third and fourth generation parenterally administered cephalosporins (ceftazidime, cefepime, piperacillin/tazobactam), monobactams (aztreonam), carbapenems (imipenem, meropenem and doripenem), novel β-lactam/β-lactamase inhibitor combinations (ceftolozane/tazobactam, ceftazidime/avibactam, imipenem/cilastatin/relebactam, meropenem/vaborbactam)
Daptomycin	
Oxazolidinones (e.g., linezolid)	
Macrolides (e.g., azithromycin)	
Lincosamides (e.g., clindamycin)	
Streptogramins (e.g., quinpristin-dalfopristin)	
Rifampin	
Trimethoprim-sulfamethoxazole	***Fluoroquinolones:*** ciprofloxacin, levofloxacin, moxifloxacin, delafloxacin
Tetracycline	
Aminopenicillins	***Aminoglycosides, neoglycosides:*** gentamicin, tobramycin, amikacin, plazmomicin
Aminopenicillin/β-lactamase-inhibitor combinations	
I–II. generation cepalosporins	***Polymyxins:*** colistin
Orally administered III generation cephalosporins	

**Table 3:** Novel and emerging therapeutic alternatives in *P. aeruginosa* [118,146,157,249,250,251,252,253,254,255,256,257,258,259,260,261,262,263,264,265,266,267,268,269,270].

**Emerging therapeutic strategy**	Description (when relevant)
**Novel antibiotics, antibiotic combination therapy**	Ceftolozane/tazobactam, ceftazidim/avibactam, imipenem/cilastatin/relebactam, meropenem/vaborbactam, plazmomicin, delafloxacin
**Existing drugs in novel formulations**	Nebulized or liposomal tobramycin, levofloxacin, aztreonam lysine, fosfomycin, colistin, and lyposimal used to treat *P. aeruginosa* in the lungs of CF patients
**Phage therapy, endolysins**	Bactericidal, highly specific to target bacteria without affecting the commensal bacteria, effective against MDR isolates, synergistic activity with antibiotics, may penetrate dense biofilms. Endolysins: they degrade the bacterial peptidoglycan from the inside of the cell during the lytic cycle of phages.
**Siderophores, iron chelation**	Perturbation of irom metabolism has been proposed as an emerging therapeutic strategy. Gallium (Ga^3+^): clinical trials include iv. gallium-nitrate (GaNite) and co-encapsulation of Ga-gentamicin in CF patients. The exact mechanism of action for Ga is still poorly understood. Some studies propose that Ga interferes with iron (Fe) uptake, Fe metabolism, and inhibits the function of Fe-containing respiratory enyzmes; however, this explanation was deemed unstatisfactory, as most compounds affecting Fe-metabolism or acting through chelation are bacteriostatic, while Ga has rapid bactericidal activity. Newer studies suggest that Ga treatment acts through the generation of ROS and the inhibition of antioxidant defence mechanisms in bacteria.
**Lectin inhibition**	Inhibition of LecA/LecB binding to lung epithelial cells.
**Quorum sensing (QS) inhibition, virulence inhibition**	Inhibition of signal molecule synthesis or sensing, which may hinder bacteria from adapting to diverse ecological niches, evading the immune system and producing virulence factors. Virulence inhibitors may “disarm” bacteria; therefore, they will not be able to induce their characteristic pathologies in vivo. In addition, as QS and virulence inhibitors do not target essential cellular components (which leads to high levels of selection pressure and the emergence of resistant mutants), it is unlikely that the host microbiome will be affected or that rapid resistance against these agents will occur.
**Efflux pump inhibitors**	
**Antimicrobial peptides (AMPs)**	
**Photodynamic therapy**	
**Vaccine development**	
**Nanoparticles (NPs)**	
**Monoclonal antibodies**	
**Conjugates**	
**Natural compounds, essential oils**	

## Data Availability

No new data were created or analyzed in this study. Data sharing is not applicable to this article.
